# An Overall View of the Functional and Structural Characterization of Glucose-6-Phosphate Dehydrogenase Variants in the Mexican Population

**DOI:** 10.3390/ijms241612691

**Published:** 2023-08-11

**Authors:** Beatriz Hernández-Ochoa, Daniel Ortega-Cuellar, Abigail González-Valdez, Víctor Martínez-Rosas, Laura Morales-Luna, Miriam Abigail Rojas-Alarcón, Montserrat Vázquez-Bautista, Roberto Arreguin-Espinosa, Verónica Pérez de la Cruz, Rosa Angélica Castillo-Rodríguez, Luis Miguel Canseco-Ávila, Abraham Vidal-Limón, Saúl Gómez-Manzo

**Affiliations:** 1 Laboratorio de Inmunoquímica, Hospital Infantil de México Federico Gómez, Secretaría de Salud, Mexico City 06720, Mexico; beatrizhb_16@comunidad.unam.mx; 2 Laboratorio de Nutrición Experimental, Instituto Nacional de Pediatría, Secretaría de Salud, Mexico City 04530, Mexico; dortegadan@gmail.com; 3 Departamento de Biología Molecular y Biotecnología, Instituto de Investigaciones Biomédicas, Universidad Nacional Autónoma de México, Mexico City 04510, Mexico; abigaila@biomedicas.unam.mx; 4 Laboratorio de Bioquímica Genética, Instituto Nacional de Pediatría, Secretaría de Salud, Mexico City 04530, Mexico; ing_vicmr@hotmail.com (V.M.-R.); lauraeloisamorales@ciencias.unam.mx (L.M.-L.); mrm.roa26@gmail.com (M.A.R.-A.); montsevazquez97@gmail.com (M.V.-B.); 5 Programa de Posgrado en Biomedicina y Biotecnología Molecular, Escuela Nacional de Ciencias Biológicas, Instituto Politécnico Nacional, Mexico City 11340, Mexico; 6 Posgrado en Ciencias Biológicas, Universidad Nacional Autónoma de México, Mexico City 04510, Mexico; 7 Departamento de Química de Biomacromoléculas, Instituto de Química, Universidad Nacional Autónoma de México, Mexico City 04510, Mexico; arrespin@unam.mx; 8 Neurobiochemistry and Behavior Laboratory, National Institute of Neurology and Neurosurgery “Manuel Velasco Suárez”, Mexico City 14269, Mexico; veped@yahoo.com.mx; 9 CICATA Unidad Morelos, Instituto Politécnico Nacional, Boulevard de la Tecnología, 1036 Z-1, P 2/2, Atlacholoaya 62790, Mexico; racastillo@ipn.mx; 10 Facultad de Ciencias Químicas, Campus IV, Universidad Autónoma de Chiapas, Tapachula City 30580, Mexico; cansecoavila@gmail.com; 11 Red de Estudios Moleculares Avanzados, Clúster Científico y Tecnológico BioMimic®, Instituto de Ecología A.C. (INECOL), Carretera Antigua a Coatepec 351, El Haya, Xalapa 91073, Mexico; abraham.vidal@inecol.mx

**Keywords:** G6PD variant, G6PD deficiency, G6PD activity, hemolytic anemia, molecular screening, neonatal jaundice

## Abstract

Glucose-6-phosphate dehydrogenase (G6PD) deficiency, affecting an estimated 500 million people worldwide, is a genetic disorder that causes human enzymopathies. Biochemical and genetic studies have identified several variants that produce different ranges of phenotypes; thus, depending on its severity, this enzymopathy is classified from the mildest (Class IV) to the most severe (Class I). Therefore, understanding the correlation between the mutation sites of G6PD and the resulting phenotype greatly enhances the current knowledge of enzymopathies’ phenotypic and genotypic heterogeneity, which will assist both clinical diagnoses and personalized treatments for patients with G6PD deficiency. In this review, we analyzed and compared the structural and functional data from 21 characterized G6PD variants found in the Mexican population that we previously characterized. In order to contribute to the knowledge regarding the function and structure of the variants associated with G6PD deficiency, this review aimed to determine the molecular basis of G6PD and identify how these mutations could impact the structure, stability, and function of the enzyme and its relation with the clinical manifestations of this disease.

## 1. Introduction

Enzymopathies are defined by a decrease in or the absence of the activity of an enzyme and are considered a particular group of innate errors of metabolism. An enzyme’s low or null catalytic activity is commonly caused by mutations in DNA. In general, these mutations lead to the modification of enzymatic activity, in part due to alterations in the native protein structure, the loss of catalytic activity, or by affecting the binding of its essential cofactors, resulting in different clinical manifestations. When an enzymopathy affects the red blood cells (RBCs), it is known as an erythro-enzymopathy, and its clinical manifestations include chronic non-spherocytic hemolytic anemias (CNSHAs) with different levels of severity [1,2]. To date, these erythro-enzymopathies have been reported in different metabolic pathways, such as the pentose phosphate pathway (PPP), glycolysis pathway [3], and glutathione metabolism pathway [4].

Glucose-6-phosphate dehydrogenase (G6PD) deficiency is an erythro-enzymopathy that has been recognized as the most common cause—through an X-linked hereditary genetic defect due to mutations in the *G6PD* gene—of CNSHA, which affects nearly 500 million people worldwide. G6PD deficiency leads to a defect in the PPP in red blood cells [5]. G6PD plays a critical role in RBCs’ metabolism because erythrocytes rely solely on PPP to generate sufficient molecules of reduced nicotinamide adenine dinucleotide phosphate (NADPH), the concentration of which in human RBCs has been reported to range from 16 to 44.9 μM [6,7]. For the obtention of the cellular NADPH pool, G6PD catalyzes the oxidation of glucose-6-phosphate (G6P) to glucose-6-phosphogluconate with the concomitant production of one molecule of NADPH, which plays an essential role in redox homeostasis and is used in RBCs as a substrate for two enzymes: glutathione reductase (GR) and thioredoxin reductase (TR). These enzymes are crucial to defend against reactive oxygen species (ROS) [8,9,10,11,12,13]. G6PD-deficient RBCs possess decreased NADPH production and, therefore, altered cellular redox homeostasis and, ultimately, increased extravascular hemolysis, which is triggered by exogenous agents such as acute infections, drugs, or food. In RBCs, the impairment of PPP can result in various degrees of hemolytic anemia and the patient may suffer from various clinical manifestations.

**Figure 1:**
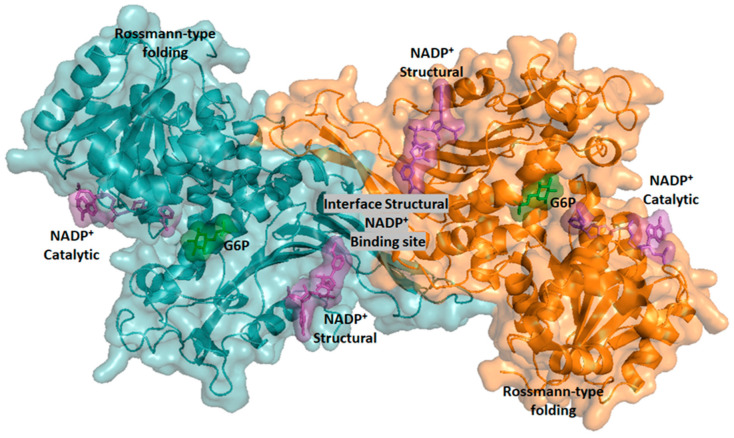
Crystallographic structure of human glucose-6-phosphate dehydrogenase (G6PD) dimer (PDB entries 2BHL and 2BH9) showing the NADP^+^ binding (purple molecular surface) at the structural and coenzyme sites and the G6P site (green molecular surface). The two monomers are shown in pale cyan and dark orange.

G6PD is a cytosolic enzyme, highly conserved through evolution, that is present in all forms of life, from prokaryotes to animals and plants [14,15,16,17]. Structurally, the human G6PD enzyme is found in monomer, dimer, and tetramer forms; however, only the dimeric form is catalytically functional, whereas interconversion between the other three forms is critical for its activity [18]. Each monomer has a binding site for G6P and catalytic NADP^+^ in addition to a structural binding site for NADP^+^ (Figure 1). Any alteration in these domains produced by genetic mutations can produce differences in disease severity for the patient; therefore, it is important to analyze and compare the structural and functional parameters observed in G6PD variants to help comprehend the consequences of each mutation [19].

To date, molecular screening has reported 231 mutations of the *G6PD* gene worldwide, generating 230 protein variants [20,21,22,23]. The mutations have been found mainly in the coding regions and are buried in the enzyme, producing functionally deficient G6PD variants [22]. Of the mutations reported, 198 variants are single missense mutations (85%), causing a single amino acid substitution; double and triple mutations are found with lower frequency (8.2%, 19 variants). In addition, 11 in-frame deletions, 1 nonsense mutation, and 2 intronic mutations were found, which indicates that this disease is heterogeneous [20,21,22,23].

## 2. G6PD Deficiency in Mexico

In Mexico, the study of G6PD deficiency was initiated by Lisker in the 1960s with clinical, hematological, genetic, and population studies [24,25]. It was not until the 1990s, with the advent of molecular biology techniques, that it became possible to reach a diagnosis that could recognize the mutations with certainty [26,27]. In Mexico, in the year 2000, a prevalence of G6PD deficiency of 0.77% was calculated by an open population cohort of 1938 subjects; in 2002, in a study carried out with a group of 4777 individuals from the general population, the prevalence was 0.71% [28]. In the latter study, it was observed that the frequency of G6PD deficiency varied between 0.39% and 4.09% in the different regions of the country. In 2018, Maldonado-Silva et al. conducted a study to describe the values of G6PD enzymatic activity quantified through neonatal screening, reporting a prevalence of 4.26% from a cohort of more than a million samples [29]. The highest prevalence was found in the states of Veracruz (21%), Nuevo León (20%), and Tabasco (15%). This prevalence coincides with that published for Mexico (0.39–4.09%) [27] and is similar to that reported in China (4.2–4.5%) [30,31]. Thus, of the 231 variants reported worldwide [23], 21 variants have been found and reported in the Mexican population (Table 1), and some of them have been described as unique for the Mexican people.

## 3. Diagnostic and Molecular Genetic Characteristics of Variants in Mexico

**Figure 2:**
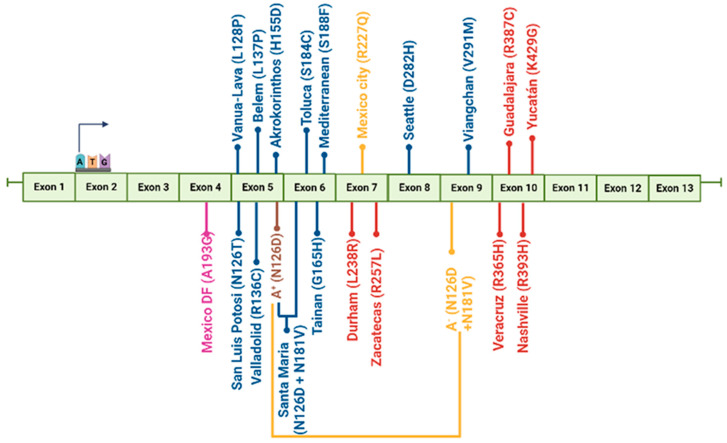
Schematic representation of G6PD mutations present in Mexican populations. The exons are represented as green-colored boxes. The Class I mutations are represented as red circles, Class II as blue circles, Class III as yellow circles, and the unclassified mutations as pink circles.

As previously mentioned, the 21 natural G6PD mutants identified in Mexico, which include single-nucleotide substitutions (missense variants) to double mutants, are distributed throughout several parts of the country. The classification of each variant, in the Mexican population, indicates that six are Class I, eleven are Class II, three are Class III, and one is unclassified. The Class I variant, G6PD Durham, was detected in a patient aged 35 years with antecedents of hemolytic anemia since the age of 5 years and with blood transfusions required several times. This variant involves the substitution of adenine for a guanine (A → G) nucleotide (nt) at position 713 (exon 7), resulting in a change from Lys to Arg in amino acid residue 238 [32,50] (Figure 2). Class I G6PD Zacatecas was detected in a twelve-year-old boy from Zacatecas State who had antecedents of neonatal jaundice and hemolytic crisis during the first nine years of life, requiring a blood transfusion. The variant involves the substitution of guanine for thymine (G → T) at nt 770 (exon 7), and the mutation results in the replacement of amino acid Arg by Leu 257 (Arg → Leu) [32] (Figure 2). The Class I G6PD Veracruz mutant was isolated in blood samples from anonymous patients from the state of Veracruz, located on exon 10, and the replacement of Arg by His 365 (Arg → His) [32]. This mutation demonstrated 15% of the activity that it would demonstrate in a healthy person. The Class I G6PD Guadalajara variant was detected in a three-year-old boy born in Guadalajara, who presented with neonatal jaundice, hemoglobinuria, and CNSHA, requiring blood transfusions. This variant is characterized by the substitution of cytosine by thymine (C → T) at nt 1159 (exon 10), which results in a change in Arg to Cys 387 (Arg → Cys) [33]. Finally, the Class I G6PD Yucatan (nt 1285 A→G, Lys429Asp) and G6PD Nashville variants (nt 1178 G → A, Arg393His) are located in exon 10 and were detected in anonymous blood samples from blood donors [28,32,51].

Regarding Class II variants, nine mutations have been found in the Mexican population. G6PD San Luis Potosi was detected in an anonymous blood sample from San Luis Potosi State. This mutant is characterized by a single-nucleotide substitution of adenine for thymine (A → T) at nt 376 (exon 5), which results in the substitution of Asn by the Tyr 126 (Asn → Tyr) amino acid residue [48] (Figure 2). The mutation in this variant is localized in the same position as the Class III G6PD A^+^ variant (nt 376). The G6PD Santa Maria variant is a double mutant that involves a substitution in exon 5 of A → G at nt 376 and an A → T transversion at nt 542, causing a change from Asn to Asp 126 (Asn → Asp) and Asp to Val 181 (Asp → Val) amino acid residues. The Santa Maria variant is related to hemolysis after the ingestion of broad beans [46,47] (Figure 2). The G6PD Vanua Lava variant is characterized by a mutation at nt 383 (T → C), at amino acid 128 (Leu → Pro), and at exon 5 [36,52] (Figure 2). The presence of the G6PD Vanua Lava variant in Mexico might be explained by population flow. There are historical antecedents of the arrival of slaves from several regions of Southeast Asia, departing from Manila and arriving at Acapulco during colonialism [28]. The G6PD Valladolid mutation (nt 406 C → T, Arg136Cys) was found in exon 5 and is associated with favism, which is the second most frequent haplotype found in Mexican Mestizos [37]. The G6PD Belem, G6PD Acrokorinthos, and G6PD Mediterranean variants were detected in the Mexican population by Vela-Amieva et al. [53] through newborn screening of Mexican children. The G6PD Belen mutation was found in one patient who was asymptomatic during the neonatal period and showed G6PD residual activity of 12% compared to a normal patient. The G6PD Akrokorinthos mutation was found in non-hospitalized patients with neonatal jaundice (NNJ), which is one of the clinical manifestations of G6PD deficiency, and 37% of G6PD residual activity was detected. Additionally, the G6PD Mediterranean mutation was detected in a patient who required hospitalization during the neonatal period and showed 1.2% of residual activity. Alcantara-Ortigoza et al. [39] described an unreported missense variant (Ser184Cys), which was named “Toluca”, and the extremely rare Gln195His, or “Tainan”, variant, which was previously described in the Taiwanese population as a Class II variant. The G6PD Toluca mutation (nt 551C → G, Ser184Cys) was identified in two hemizygous nonrelated newborn G6PDd males, while the extremely rare G6PD Tainan mutation (nt 585G → C, Gln195His) was identified in a third hemizygous newborn G6PDd. The G6PD Seattle variant has a single-nucleotide substitution of guanine by cytosine (G → C) at nt 844 (exon 8), produced as a result of the substitution of Asp to the His 282 (D → H) amino acid residue. This mutation was detected in a family of G6PD-deficient Caucasian subjects without CNSHA [41]. Interestingly, this mutation was also found in Mexican populations from different regions of Mexico. Finally, G6PD Viangchan was first reported in a Laotian immigrant (from the city of Viangchan) from Calgary, Canada, and was characterized by severe enzyme deficiency [54]. G6PD Viangchan is characterized by a G → A substitution in nt 871 at exon 9, with a resultant change in the amino acid Val to Met 291 (Val → Met) (Figure 2). This mutant is a polymorphic Southeast Asian variant associated with specific ethnic groups in tropical Asia [34,55,56,57].

In addition, three natural Class III variants have been detected in the Mexican population. The single natural G6PD A+ variant involves a change in nucleotide (nt) 376 of adenine by guanine (A → G), resulting in the substitution of asparagine by an aspartic acid (Asn → Asp) amino acid residue in the 126 position. This variant is related to a form of asymptomatic G6PD deficiency [28,48]. The G6PD Mexico City variant (nt 680 G → A, Arg227Gln) has only been found in Mexico, and the mutation is located in exon 7 [26].

The double mutant G6PD A− (A376G/T968C) involves the mutations G6PD A+ and G6PD Nefza (Leu to Pro 323) [58] (Figure 2). This double mutant is classified as a Class III variant because the patients showed episodes of hemolysis triggered by infections, drugs, or food [59]. Furthermore, it was observed that patients with this double mutant showed residual glucose-6-phosphate dehydrogenase activity of around 10–20% [60,61].

Finally, the unclassified G6PD Mexico DF was detected in male volunteer blood donors in a genetic and molecular study of G6PD deficiency in Northern Mexico [49]. This mutation was characterized by a nucleotide change in position 193 of adenine by guanine (A → G) in exon 4, producing a change in Ala by Thr (Ala → Thr) in the amino acid residue 65.

## 4. Mutations of G6PD Variants Affect Its Activity in Different Manners

**Figure 3:**
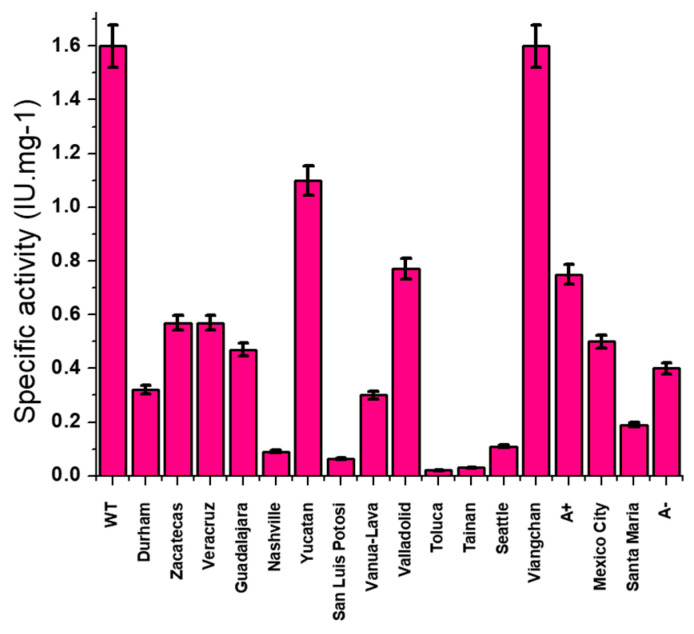
Overexpression of G6PD variants in a heterologous system (*Escherichia coli* BL21(DE3)Δzwf::kanr^r^). Specific activity measured in the crude extract was used as indicative of soluble recombinant protein. The crude extract was measured at 340 nm and 25 °C to calculate specific activity based on the concentration of protein, using the reaction standard mixture (100 mM Tris–HCl buffer at pH 8.0, 3 mM MgCl_2_, 1 mM of G6P, and 1 mM NADP^+^). The figure was created using the data found in references [10,14,39,62,63,64,65].

**Figure 4:**
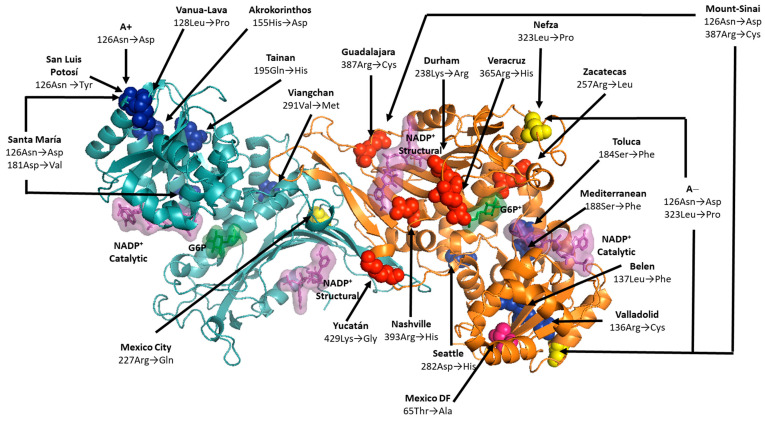
Localization of the mutations on the G6PD structure. The amino acid residues replaced in the Class I G6PD (red), Class II (blue), Class III (yellow), and unclassified (pink) variants are shown in spheres.

To explore the underlying characteristics at the molecular level of the G6PD variants present in the Mexican population and their relationships with clinical manifestations, our working group constructed 19 of the 21 G6PD variants present in the Mexican population [10,14,39,62,63,64,65]. The 16 single clinical mutants and the two double mutants were created by site-directed mutagenesis corroborated by bidirectional DNA sequence analysis. For all the G6PD variants, the specific activity of G6PD enzymes was time-dependent, with maximal specific activity at 18 h in most cases, with the exception of the G6PD Veracruz (Class I), Seattle, and Santa Maria variants (Class II), with an optimal expression time of 12 h [14,62]. As expected, the expression of Class I G6PDs was less than that of the WT-G6PD enzyme (i.e., 1.6 IU·mg^−1^) [10] (Figure 3). Specific activities from 0.09 IU·mg^−1^ to 0.57 IU·mg^−1^ in the crude extract were obtained, which represents a 2.8- to 17-fold decrease in the specific activity regarding the WT-G6PD enzyme (i.e., 1.6 IU·mg^−1^) [10,14,39,62,63,64,65]. Class II–III variants’ specific activities from 0.11 IU·mg^−1^ to 1.16 IU·mg^−1^ in the crude extract were obtained. It is interesting to note that although the mutations were in different parts of the three-dimensional structure of the WT-G6PD protein (Figure 4), the expression of soluble protein measured by specific activity was lower than in WT-G6PD [10,14,39,62,63,64,65].

The recombinant G6PD variants with purity greater than 95% were obtained for each mutant [10,14,39,62,63,64,65]. Approximately 2 mg of almost pure protein for all the variants was obtained, except for the Class II G6PD San Luis Potosi, Vanua Lava, Toluca, Tainan, Seattle, and unclassified Mexico DF variants [14,39,63,65], where the total protein ranged from 0.4 to 0.6 mg of pure protein, suggesting that a decrease in the stability of the protein in each one of the mutants, compared to the WT-G6PD protein, was provoked by the single mutations on the 3D structure (Figure 4). Furthermore, independently of this, the mutations were located in different regions of the three-dimensional structure and were distant from the active site or the structural NADP^+^ region. All G6PD variants exhibited a negative effect on expression and purification, which could be related to the lower stability of the variants in the erythrocyte, which causes different clinical manifestations (Figure 4) [10,14,39,62,63,64,65].

## 5. Steady-State Kinetic Parameters of G6PD Variants

To understand how different mutations cause varying degrees of enzyme deficiency, a detailed study of the structural stability and catalytic activity of G6PD variants was required. The steady-state kinetic parameters of the 19 G6PD variants present in the Mexican population were determined. The Class I G6PD variants presented a greater loss of affinity for both physiological substrates (G6P and NADP^+^) [10,14,62,63,65]. It is interesting to note that among the Class I variants, the G6PD Zacatecas Arg257Leu mutation was close to the G6P binding region and was the most affected variant with respect to the affinity of substrates, demonstrating a G6P reduction of almost 35%, while, for the NADP^+^ substrate, it decreased by 26% (Table 2) [63]. The Guadalajara and Nashville variants demonstrated the next largest decreases [10,65], while the G6PD Durham, Veracruz, and Yucatan variants showed the smallest changes in the affinity of substrates [10,14,62]. Regarding the catalytic constant (*k*_cat_), all G6PD variants showed a lower value compared with the WT-G6PD. Among the Class I G6PD variants, we observed a loss of 40% to 95% in *k*_cat_ values. The Guadalajara (Arg387Cys) variant has been associated with a severe manifestation of the disease. Martinez-Rosas et al. [65] reported that the G6PD Guadalajara variant lost more than 90% of catalysis (*k*_cat_ = 25.9 s^−1^) compared to the WT-G6PD enzyme. Furthermore, the specificity constants for G6P and NADP^+^ substrates decreased approximately 40-fold for G6P (*k*_cat_/*K*_mG6P_ 0.15 × 10^6^) and 102-fold for NADP^+^ (*k*_cat_/*K*_mNADP+_ 0.37 × 10^6^) (Table 2) [65]. The steady-state kinetic parameters determined for G6PD Guadalajara are in agreement with the clinical manifestations reported in a three-year-old boy born suffering from neonatal jaundice, hemoglobinuria, and chronic non-spherocytic hemolytic anemia, requiring blood transfusions [33]. The second most affected mutant was G6PD Zacatecas, where 75% catalysis was recorded and specificity constants for G6P and NADP^+^ substrates decreased approximately 11-fold for G6P (*k*_cat_/*K*_mG6P_ 0.52 × 10^6^) and 15-fold for NADP^+^ (*k*_cat_/*K*_mNADP+_ 2.41 × 10^6^) (Table 2) [63].

Regarding Class II G6PD variants, a loss of 40% to 60% in the catalytic constant (*k_cat_*) for the Vanua Lava, Valladolid, Viangchan, and Santa Maria variants was observed [10,62,63]. However, among the Class II variants, the G6PD San Luis Potosi, Toluca, Tainan, and Seattle variants were the most affected, with a loss of 95% in *k*_cat_ [14,39,65]. In particular, we observed a loss in the specificity constants for the NADP^+^ substrate (*k*_cat_/*K*_mNADP+_), where a 135-fold (0.28 × 10^6^), 90-fold (0.42 × 10^6^), 50-fold (0.75 × 10^6^), and 31-fold (1.2 × 10^6^) decrease was observed for the San Luis Potosi, Tainan, Toluca, and Seattle variants, respectively (Table 2) [14,39,65]. It is important to mention that although these variants were classified as Class II mutants according to patients’ hematological parameters, the steady-state kinetic parameters and the loss of catalysis were similar to the values obtained for Class I mutants [10,14,62,63,65]. Finally, the steady-state kinetic parameters of Class III G6PD Mexico City were reduced by approximately 50% when compared with that of the WT-G6PD enzyme [10], while the double mutant Class III G6PD A− (Asn126Asp + Leu323Pro) and unclassified Mexico DF variants showed a loss of 85% of catalytic activity in terms of the WT-G6PD enzyme [14,64]. The loss of catalysis (*k_cat_*), the affinity for both physiological substrates (*K*_mG6P_ or K_mNADP_^+^), and the specificity constants (*k*_cat_/*K*_m_) for all the G6PD variants were in accordance with the severity of the clinical manifestations, where Class I variants demonstrated the most severe manifestation of the disease, chronic non-spherocytic hemolytic anemia. Furthermore, these alterations in the steady-state kinetic parameters of the WT-G6PD enzyme suggest that the variants, because they are synthesized, are kinetically defective, due to the fact that they produce alterations in the steady-state kinetic parameters. This may explain the low production of the reduced form of NADPH, leading to difficulty in maintaining redox homeostasis in red blood cells and, ultimately, increased extravascular hemolysis in patients [62,63,66]. Finally, these data suggest that the catalytic efficiency is affected differentially by these mutations and that one of the variables involved in the degree of damage in the loss of catalysis and affinity for the two physiological substrates is the location of the mutation in the tertiary structure (Table 2) [10,14,39,62,63,64,65].

**Table 2:** Summary of kinetic parameters of WT-G6PD and variants present in Mexico.

G6PD	Amino Acid Substitution	Class	*k*_cat_ (s^−1^)	K_mG6P_ (µM)	K_mNADP_^+^ (µM)	*k*_cat_/K_mG6P_ (s^−1^ M^−1^)	*k*_cat_ K_mNADP_^+^ (s^−1^ M^−1^)	References
WT			233	38.5	6.2	6.0 × 10^6^	37.8 × 10^6^	[10]
Durham	Lys238Arg	I	71	24.77	6.96	2.85 × 10^6^	10.2 × 10^6^	[62]
Zacatecas	Arg257Leu	I	58	111	24	0.52 × 10^6^	2.41 × 10^6^	[63]
Veracruz	Arg365His	I	50.6	18.7	31.8	2.7 × 10^6^	1.6 × 10^6^	[14]
Guadalajara	Arg387Cys	I	25.9	98.8	41.4	0.15 × 10^6^	0.37 × 10^6^	[65]
Nashville	Arg 393His	I	119	90.6	31.2	1.3 × 10^6^	3.8 × 10^6^	[10]
Yucatan	Lys429Gly	I	138	39.9	6.4	3.5 × 10^6^	21.7 × 10^6^	[10]
San Luis Potosi	Ans126Ty	II	10.4	43.7	11.9	1.3 × 10^6^	0.28 × 10^6^	[65]
Vanua Lava	Leu128Pro	II	142	34	18	4.1 × 10^6^	7.88 × 10^6^	[63]
Valladolid		II	96	21.5	3.6	4.4 × 10^6^	26.2 × 10^6^	[10]
Acrokorinthos	Asn126Asp + His155Asp	II	19.3	38.1	8.4	0.5 × 10^6^	2.3 × 10^6^	[67]
Toluca	Ser184Cys	II	8.2	51.9	10.9	1.3 × 10^5^	0.75 × 10^6^	[39]
Tainan	Gln195His	II	4.7	51.1	11.1	0.7 × 10^5^	0.42 × 10^6^	[39]
Seattle	Asp282His	II	16.2	24.8	13.9	0.6 × 10^6^	1.2 × 10^6^	[14]
Viangchan	Val291Met	II	145	42	17	3.45 × 10^6^	8.52 × 10^6^	[63]
Santa Maria	Asn126Asp Asp181Va	II	71	15.35	9.06	4.62 × 10^6^	7.83 × 10^6^	[62]
A− (968)	Asn126 Asp Leu323 Pro	III	35.8	33.8	14.3	1.1 × 10^6^	2.5 × 10^6^	[64]
Mexico City	Arg227Gln	III	182	24.9	9.1	7.3 × 10^6^	19.1 × 10^6^	[10]
A+	Asn126Asp	IV	114	56.4	12.9	2.0 × 10^6^	8.7 × 10^6^	[64]
Mexico DF	Thr65Ala	NR	34.6	92.8	26.6	0.4 × 10^6^	1.3 × 10^6^	[14]

NR: class not reported. The kinetic parameters of the G6PD variants were determined spectrophotometrically at 25 °C, monitoring the reduction of the NADP^+^ substrate to absorbance of 340 nm. Standard activity assay was performed in buffer T (Tris–HCl 0.1 mM, pH 8.0, and MgCl_2_ 3 mM).

## 6. Effect of Mutation on the Stability of the G6PD Variants

Different approaches are utilized to determine the stability of proteins, such as thermal inactivation, guanidine hydrochloride, and trypsin digestion. We previously performed these methods to evaluate the effect of mutation on the stability of the G6PD variants, which could be the most frequent deleterious effect caused by mutations and could explain the clinical manifestations of G6PD deficiency. Next, we summarize our previous findings.

### 6.1. Thermal Inactivation Assays

**Figure 5:**
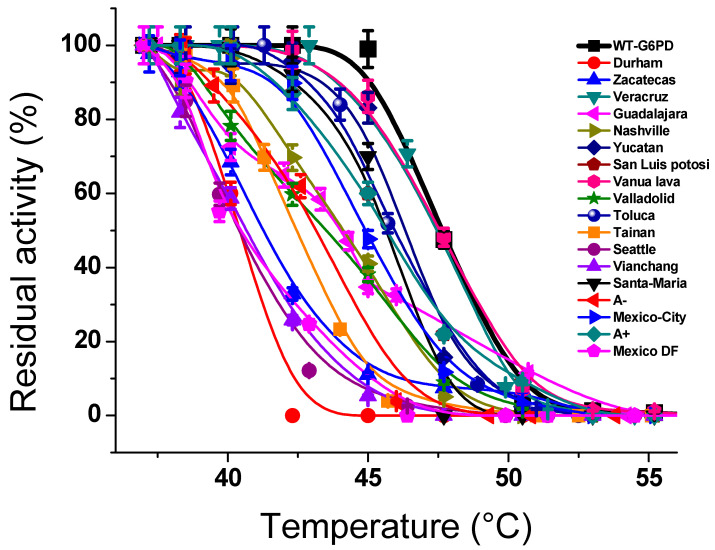
Representative thermal inactivation assays of recombinant G6PD variants in the absence of NADP^+^. In all cases, 200 ng of total protein was used. The activity was measured with the reaction standard mixture (100 mM Tris–HCl buffer at pH 8.0, 3 mM MgCl_2_, 1 mM of G6P, and 1 mM NADP^+^). Residual activity was expressed as a percentage of the activity for the same sample incubated at 37 °C. The figure was created using the data found in references [10,14,39,62,63,64,65].

Thermal unfolding methods are commonly used to assess the impact of different mutations on the protein structure, stability, and activity of G6PD [10,14,62,63,64,65,66,68,69]. In the thermal inactivation assay, the *T*_50_ was obtained and defined as the temperature at which the enzyme lost 50% of its activity. When the WT-G6PD and the variants were incubated at different temperatures (37 to 55 °C for 20 min), all the G6PD variants were most susceptible to temperature compared with WT-G6PD (Figure 5) [10,14,39,62,63,64,65]. It is interesting to note that Class I variants, such as Durham, Zacatecas, Guadalajara, and Nashville, were the most unstable variants because these variants’ *T*_50_ values decreased by around 10 °C for the single natural mutation of the WT-G6PD enzyme [10,62,63,65]. Furthermore, it is interesting to note that these mutations were located at the structural NADP^+^ binding site positioned close to the interface, where the two subunits of each dimer were intertwined. If the correct positioning of structural NADP^+^ confers stability to the human G6PD protein, improving its resistance to denaturation by temperature, then any mutation in this site causes a loss of stability in the G6PD protein, revealing the contribution of protein instability to the clinical manifestations of the G6PD variant.

Furthermore, *T*_50_ values were calculated in the presence of different NADP^+^ concentrations, since the presence of NADP^+^ improves the structural stability of the protein, increasing the *T_5_*_0_ values. As seen in Table 3, WT-G6PD showed a *T*_50_ of 48 °C in the absence of NADP^+^, while, with 500 µM NADP^+^, the *T*_50_ value was 59 °C, demonstrating an increase of 11 °C, indicating that the union of the NADP^+^ molecule in the structural NADP^+^ binding site favors the stability of the protein and protects it at high temperatures (Table 3) [10]. It is interesting to note that most Class II–III G6PD variants showed *T*_50_ values between 40 °C and 46 °C in the absence of NADP^+^, while a stabilizing effect with 500 µM NADP^+^ was observed for the mutants, showing an increase in *T*_50_ between 8 °C and 11 °C [10,14,39,62,63,64,65]. It is important to note that although Class II–III variants demonstrated alterations in catalytic activity and a loss of affinity for both physiological substrates, the presence of NADP^+^ was found to improve the structural stability of the protein in these variants. However, the results among variants of Class II–III, such as San Luis Potosí, Seattle, and Mexico DF, were striking [14,65]. Although these variants were stable in the presence of the NADP^+^ molecule, they presented significant losses of catalytic activity and affinity for both physiological substrates compared to the WT-G6PD enzyme, indicating that the loss of activity was caused by the loss of catalysis rather than the loss of stability [14,65]. Finally, regarding the Class I variants, it was determined that the enzymes belonging to this group did not improve their stability in the presence of NADP^+^, where a shift in their *T*_50_ values between 3 °C and 4 °C was recorded [10,14,62,63,65]. It is noteworthy that mutations occurring near the structural binding site of NADP^+^ decrease the stability and catalysis of the enzyme, causing severe phenotypes such as CNSHA [10,14,62,63,64,65,66,68,69,70]. These results are in accordance with previous reports on the G6PD variants Plymouth (I), Andalus (I), Bangkok (I), Bangkok noi (I), Canton + Bangkok noi (I), Songklanagarind (II), Union (II), Canton (II), Ananindeua (II), Acrokorinthos (II), Sierra Leone (III), Asahi (III), and Union + Viangchan (II/III), where the presence of NADP^+^ improved the stability of the G6PD variants’ responses to temperature in a manner that was dependent on the NADP^+^ concentration [66,67,68,69].

### 6.2. Stability of G6PD Variants in the Presence of Guanidine Hydrochloride

**Figure 6:**
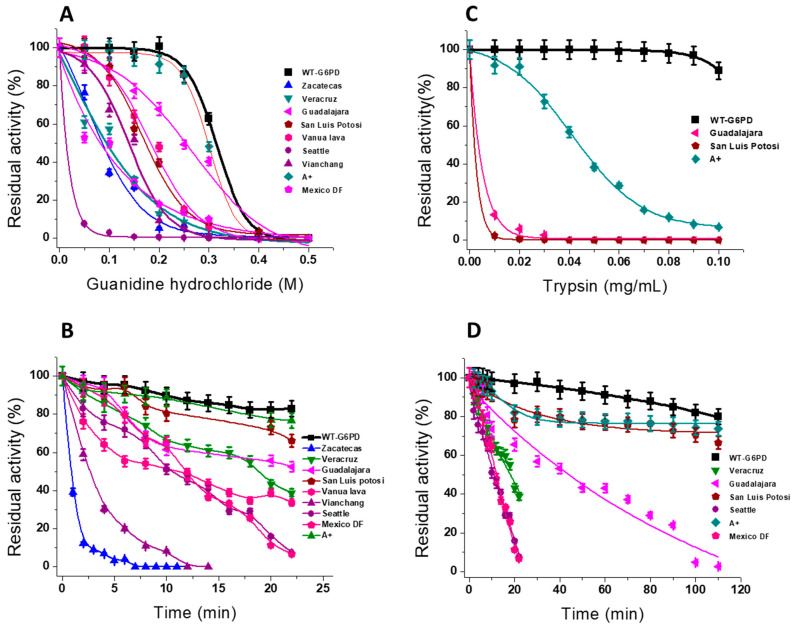
Stability of G6PD variants in presence of guanidine hydrochloride (Gnd-HCL) and trypsin digestion. (**A**) Dose–response curves in presence of different concentrations of (Gnd-HCL). All enzymes were incubated at 0.2 mg/mL in 50 mM phosphate buffer pH 7.35 in the presence of the indicated concentrations of Gdn-HCl for 2 h at 37 °C, and, subsequently, the enzymatic activity was measured. Residual activity was expressed as a percentage of the activity for the same sample measured at 25 °C without Gdn-HCl. (**B**) Inactivation of WT G6PD and the variants incubated with 0.25 M of Gnd-HCL. At indicated times, aliquots were withdrawn from samples and assayed for residual activity. (**C**) Dose–response curves in presence of different concentrations of trypsin. The G6PD proteins were adjusted to a final concentration of 0.2 mg/mL and incubated with trypsin (0.5 mg/mL) at 37 °C for 2 h. Residual activity was expressed as a percentage of the activity for the same sample measured at 25 °C without trypsin. (**D**) Inactivation of WT G6PD and the variants incubated with trypsin at 0.5 mg/mL at 37 °C. At the times indicated, the reaction was arrested by the addition of PMSF 5 mM, and the residual activity was measured under standard conditions. The figure was created using the data found in references [10,14,39,62,63,64,65].

Gdn-HCl is a chaotropic molecule used to determine the conformational stability of proteins because it affects the noncovalent interactions of the proteins and alters the tertiary structure, triggering their denaturation. Thus, the unfolding of G6PD variants during exposure to a Gdn-HCl denaturant can be used to investigate their structural stability. The residual enzyme activity was measured and expressed as a percentage of the activity for the same enzyme incubated without Gdn-HCl. All the G6PD variants analyzed with Gdn-HCl were least tolerant to Gdn-HCl treatment, as they showed a lower IC_50_ value compared to WT-G6PD [10,14,63,64,65]. As seen in Table 3, an IC_50_ value of 310 μM was reported for WT-G6PD [10], while, for Class I variants G6PD Veracruz, Zacatecas, and Guadalajara, an IC_50_ value of 80, 100, and 250 μM, respectively, was recorded [14,63,65]. Regarding the Class II San Luis Potosí, Vanua Lava, Seattle, and Vianchang variants, IC_50_ values of 170, 200, 13, and 150 μM, respectively, were reported [14,63,65]. The Seattle variant was the least tolerant, even though the mutation was not close to the catalytic site or the structural binding site of NADP^+^ [14]. An IC_50_ value of 200 μM was recorded for the Class IV G6PD A+ variant (Figure 6A) [64], while the unclassified Mexico DF variant was more susceptible to Gdn-HCl denaturation, with an IC_50_ of 80 μM, compared to G6PD-WT [14]. It is interesting to note that all the G6PD variants were low-stability enzymes with decreased structural stability when compared with the WT-G6PD enzyme [10,14,63,64,65]. Consistent with our structural analyses described above, the G6PD Union + Viangchan (I), Songklanagarind (II), Bangkok noi (I), Union (II), and Canton (II) variants were found to be the least stable enzymes, indicating that these variants were structurally unstable [71].

The G6PD variants were incubated with 0.25 M of Gdn-HCl and stability was evaluated by time-course inactivation [10,14,63,64,65]. As seen in Figure 6B, compared to the WT-G6PD enzyme, all the G6PD variants were more unstable in time-course inactivation. The Zacatecas and Vianchang variants were the most structurally unstable proteins [63], followed by the Mexico DF and Seattle variants [14]. These results are in agreement with thermal stability analyses in which the Zacatecas, Guadalajara, and Mount Sinai mutants were less stable and relaxed in the active site of the WT-G6PD enzyme.

### 6.3. Trypsin Digestion

Another approach to evaluating the structural instability of the proteins is using protease digestion, which imitates physiological conditions, and evaluating the enzymatic activity in vitro. To explore the unfolding/misfolding effect of G6PD mutations on the protein structure, proteolytic susceptibility assays with trypsin have been widely used. All the variants displayed high susceptibility to trypsin digestion in comparison to WT-G6PD (Figure 6C) [10,14,63,64,65]. The Class I G6PD Veracruz variant lost 60% of its activity at 25 min of incubation [14], while the G6PD Seattle and the G6PD Mexico DF variants unexpectedly lost 100% of their residual activity at the same incubation time [14]. The WT-G6PD enzyme retained greater activity (80%) at 25 min (Figure 6D) [10]. In contrast, the Guadalajara variant was more resistant to trypsin digestion: at 25 min, it retained 55% of its residual activity [65]. Finally, it is interesting to note that the Class II G6PD San Luis Potosi variant (Asn126Tyr) [65] presented the same pattern of susceptibility to trypsin digestion as the Class IV G6PD A+ (Asn126Asp) variant [64], probably due to both variants possessing a mutation in the same codon. All the G6PD variants were more susceptible to trypsin digestion compared with the WT-G6PD enzyme. Nevertheless, when some G6PD variants analyzed were incubated at the physiological concentration of NADP^+^ (10 μM), all the variants became more resistant to protease degradation, indicating that the addition of NADP^+^ produced a protective effect against trypsin digestion (Figure 6D) [10,14,64,65]. These results are in agreement with those previously reported by Boonyuen et al. [66], who studied the susceptibility to trypsin digestion for the Class II G6PD Viangchan and Class II G6PD Mahidol variants. Their research showed that the presence of NADP^+^ improved the stability against proteolysis in the aforementioned variants. This protective effect of trypsin digestion is likely be due to steric hindrance by the binding of the NADP^+^ molecule to cleavage sites for trypsin in the native WT-G6PD protein. Finally, it is interesting to note that all the G6PD variants were more sensitive to Gdn-HCl and the protease degradation of the WT-G6PD enzyme, indicating that the accelerated degradation of the G6PD variants could be caused by defective protein folding, which also usually contributes to reduced protein stability, as observed in G6PD-deficient individuals.

## 7. Structural Characterization of G6PD Variants

Because the functional analyses described above suggested that the single mutant was mainly responsible for the loss of catalytic activity and structural stability in all the G6PD variants analyzed, we aimed to determine whether the mutations had local and global effects. Based on the above, the G6PD variants were analyzed by circular dichroism (CD) and intrinsic and extrinsic fluorescence assays.

### 7.1. Circular Dichroism Analysis

**Figure 7:**
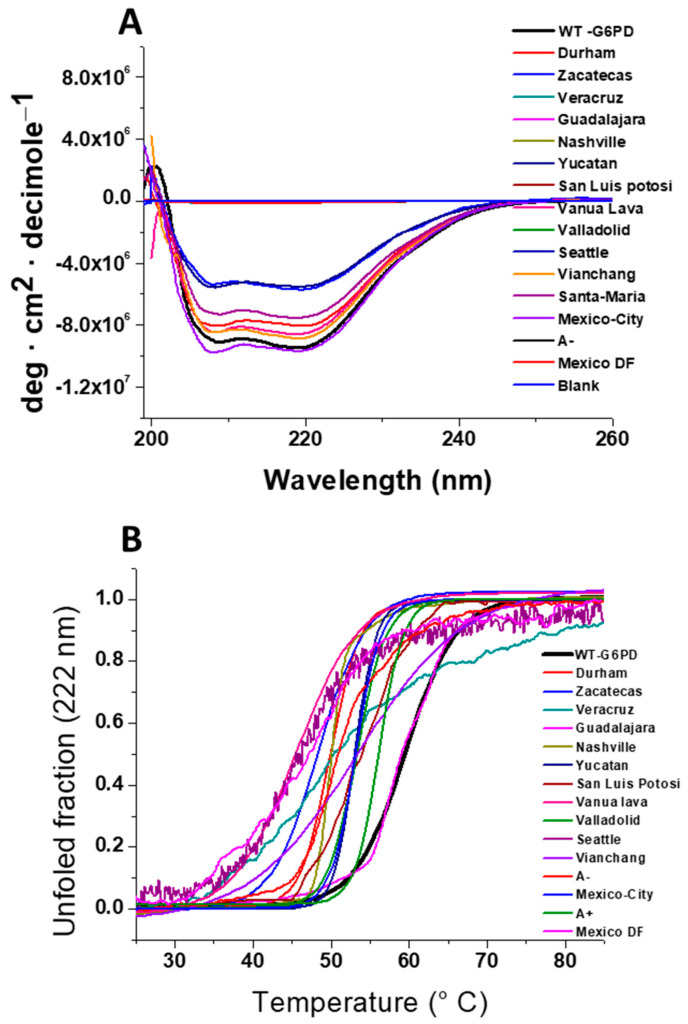
Stability analysis of recombinant human WT-G6PD and variants. (**A**) Circular dichroism analysis of G6PD variants. All the CD spectra were recorded at 25 °C. Far UV-CD spectra of the G6PD variants were detected from 200 to 260 nm at 1 nm intervals in 50 mM phosphate buffer at pH 7.35 and 25 °C. (**B**) Thermal stability analysis of recombinant G6PD variants. Thermal stability and unfolding of G6PD variants were analyzed following the changes in CD signal at 222 nm and temperature scans from 20 to 85 °C in 50 mM phosphate buffer at pH 7.35. The figure was created using the data found in references [10,14,62,63,65].

Because the G6PD variant enzymes present in the Mexican population have diminished purification yields and decreased catalytic efficiency, CD assays were employed to determine alterations in the secondary structure produced by the change in amino acid residue in each variant. As shown in Figure 7, the overall G6PD structure was maintained in the analyzed variants and WT-G6PD, with minimum negative absorption peaks at 208 and 220 nm [10,14,62,63,64,65]. However, the majority of the variants had lower signal intensities than the WT enzyme, indicating that the mutation in each variant altered the secondary structure of the protein. The CD spectra of the G6PD Veracruz, Guadalajara, San Luis Potosi, Seattle, G6PD A−, and Mexico DF variants showed greater changes in signal intensity regarding WT-G6PD (Figure 7A) [14,63,64,65]. Such intensity changes indicate that the chirality of the chromophores is modified upon mutation, which provides information about the flexibility or rigidity of secondary structure elements, as reported by Praoparotai et al. [67]. The G6PD Durham, Zacatecas, Yucatan, Vanua Lava, Vianchang, Santa Maria, and Mexico City variants presented fewer alterations in the pattern and intensity of CD spectra for WT-G6PD (Figure 7A) [10,62,63]. These results suggest that the alterations in the catalytic activity observed in these mutants were due to alterations in the secondary structure of the protein, most likely due to conformational changes at the global level of the three-dimensional structure of the protein. In addition, similar results were reported in the G6PD Mahidol, Vianchang, Vianchang + Mahidol, Bangkok (I), Bangkok noi (I), Canton + Bangkok noi (I), Songklanagarind (II), Union (II), Canton (II), Ananindeua (II), Acrokorinthos (II), Sierra Leone (III), Asahi (III), and Union + Viangchan (II/III) variants [66,67].

### 7.2. Thermal Stability Analysis of Recombinant G6PD Variants

Due to several mutants showing alterations in their secondary structures and losing catalytic activity, we also evaluated the three-dimensional alterations of each of the variants by thermal stability assays, another assay that evaluates global protein stability. As seen in Figure 7B, the global stability of all G6PD clinical variants analyzed in this review was altered in the presence of a mutation [10,14,62,63,64,65].

The effect of mutations on global stability showed that the most affected variants were the Class I G6PD Durham, Zacatecas, Veracruz, Guadalajara, and Nashville variants (loss of approximately 13 to 10 °C) [10,14,62,63,65]. It is interesting to note that the Vanua Lava and Seattle Class II G6PD variants presented a loss of global stability similar to the Class I variants [14,63]. Similar results were observed for the Bangkok (I), Viangchan (II), Mahidol (II), Union (II), Canton (II), Union + Viangchan (II/III), Viangchan + Mahidol (II/III), and Songklanagarind (II) G6PD variants, where greater alterations in thermal stability were observed [66,71]. Furthermore, in two Class I G6PD clinical mutants, G6PD Fukaya (G488S) and G6PD Campinas (G488V) [72]—where the mutations were in the vicinity of the “structural” NADP+ site—the Tm values were around 10 °C lower compared to WT G6PD.

The Class I Yucatan G6PD variant and the Class II–III San Luis Potosi, Valladolid, Viangchan, Santa Maria, and double G6PD mutants (G6PD A+ and A−) showed a loss in Tm values of around 6 °C compared to the native enzyme [10,62,63,64,65]. Similarly, Boonyuen et al. [66,71] found that Class I variants, such as the Bangkok (I) variant, were less stable than the native protein, where the value was 6 °C lower than that of the WT-G6PD enzyme. The changes observed in the structural stability of Class I–II G6PD variants indicate that all G6PD mutants are more susceptible to denaturation by temperature because these mutations make the three-dimensional (3D) structure less stable compared to WT-G6PD. This may be related to the loss of catalytic efficiency of these enzymes and could have a relationship with the clinical manifestations in patients.

### 7.3. Intrinsic Fluorescence and ANS Binding Assays

**Figure 8:**
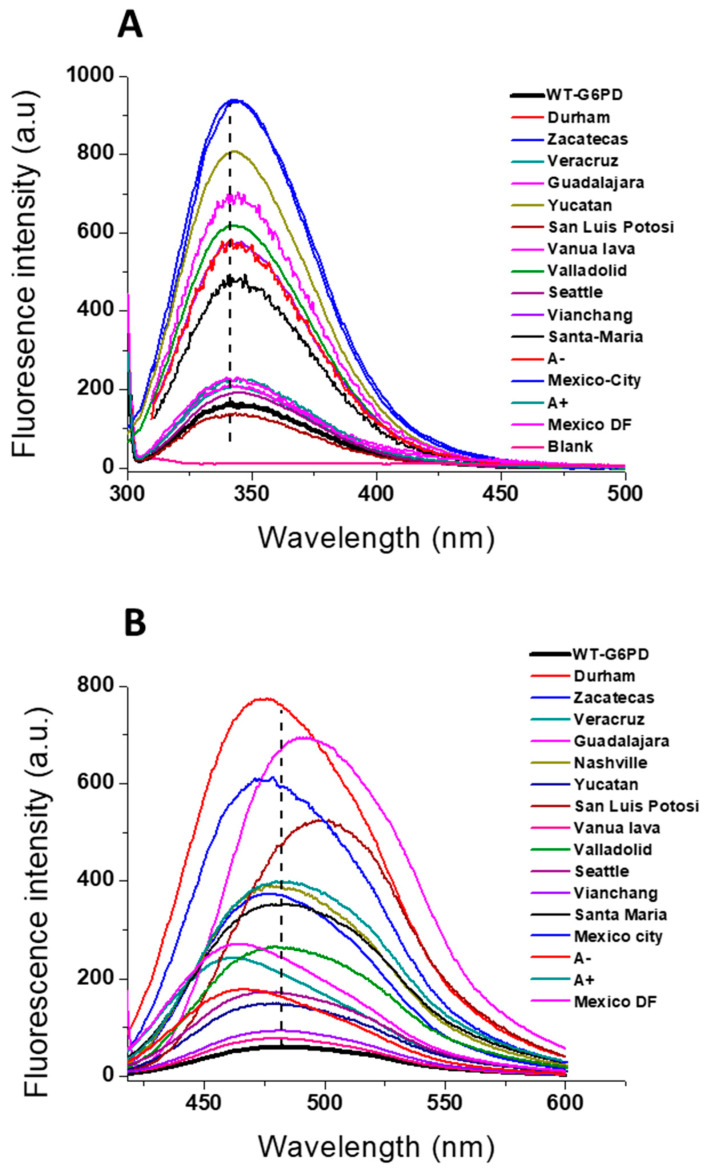
Fluorescence emission spectra of WT-G6PD and variants. (**A**) Intrinsic fluorescence spectra and (**B**) 8-anilinonaphthalene-1-sulphonate (ANS) accessibility assays. The intrinsic and extrinsic fluorescence assay of G6PD variants was performed using a final protein concentration of 0.1 mg/mL in 50 mM phosphate buffer at pH 7.35 at 25 °C. The figure was created using the data found in references [10,14,62,63,64,65].

To evaluate the effect of the single or double mutant on the three-dimensional structure of the native G6PD enzyme and determine whether the activity loss was correlated with the disruption of the protein’s structural stability, intrinsic and extrinsic fluorescence assays were carried out. Intrinsic fluorescence (IF) has been widely used to monitor the changes in the fluorescence emission maxima of the seventh tryptophan residues contained in the G6PD by a monomer in the native enzyme. As shown in Figure 8A, the fluorescence emission spectra for all variants were increased compared to WT-G6PD [10,14,62,63,64,65]. It is interesting to note that all the variants analyzed showed a fluorescence emission spectrum at a peak of 338 nm, indicating that none of the mutants showed a tendency to aggregate. Similarly, Boonyuen et al. [71] reported that three G6PD variants—G6PD Bangkok, Bangkok noi, and Songklanagarind—also showed an emission peak at around 338 nm.

The Class I G6PD variants (Zacatecas, Durham, and Veracruz) showed more alterations in the emission spectrum (two-fold compared to the WT-G6PD enzyme) [14,62,63], while the Class II–III variants (San Luis Potosi, Santa Maria, Seattle, A−, and A+) showed fewer alterations in the emission spectrum (ranging from 1.2-fold to 1.8-fold compared to the WT enzyme) [14,62,64,65]. However, the fluorescence intensity demonstrated by the G6PD Vanua Lava variant was the same as that of WT-G6PD, while the G6PD Bangkok noi, Songklanagarind, Asahi, Sierra Leone, Acrokorinthos, and Ananindeua variants showed a fluorescence intensity lower than that of the native enzyme [67,71]. It is important to note that Ghisaidoobe and Chung [73] reported that a decrease in fluorescence intensity could be attributed to electron transfer quenching by the local peptide carbonyl group or by neighboring amino acid side chains as a result of conformational changes or interactions with ligands [67,71,73]. This increase or decrease in intrinsic fluorescence intensity for all the G6PD variants suggests modifications and conformational changes in the microenvironment of the tryptophan residues, causing these tryptophan residues to be exposed to a more hydrophilic environment in the native three-dimensional structure of this protein, which has a negative effect on the catalytic activity in the clinical mutants.

Additionally, the emission fluorescence spectra of 8-anilinonaphthalene-1-sulfonate (ANS) have been shown to determine the degree of structural perturbation on the global stability caused by mutations. As seen in Figure 8B, all the G6PD variants showed an increase in extrinsic fluorescence (EI) emission spectra compared to WT-G6PD [10,14,62,63,64,65]. Similar results were reported for G6PD variants Bangkok noi (I), Bangkok (I), Canton + Bangkok noi (I), Songklanagarind (II), Union (II), Canton (II), Acrokorinthos (II), Ananindeua (II) Union + Viangchan (II/IIIAsahi (III), and Sierra Leone (III) [71,73]. These modifications in fluorescence intensity in the G6PD variants may be due to several factors that can be attributed to alterations in protein surface hydrophobicity, such as conformational changes, protein denaturation, protein unfolding, oxidative modification, and binding to some ligands [71].

Moreover, the maximal fluorescence intensity of the spectrum of ANS in WT-G6PD was different in the G6PD Durham, G6PD Veracruz, Santa Maria, A+, and Mexico DF variants because a displacement in the maximal fluorescence intensity (ANS of 10–17 nm in the G6PD variants) was observed [14,62]. It is interesting to note that this blue shift (a shift to a shorter wavelength) was also observed in the Bangkok (I), Canton + Bangkok noi (I), Songklanagarind (II), Union (II), and Canton (II) G6PD variants. In all of these, the increased fluorescence spectra and the shift to the blue region indicate that the ANS found more hydrophobic pockets and buried hydrophobic pockets in the G6PD variant enzymes, suggesting that all the G6PD mutations showed more hydrophobic regions exposed to the solvent than the WT-G6PD enzyme, producing a more relaxed 3D structure. Similar phenomena have also been observed in other G6PD variants, such as G6PD Wisconsin, Viangchan, Canton, and Mahidol [68,69].

All results analyzed in this review confirm that mutations in the G6PDs’ native protein have a strong effect on the stability of these structures, producing lower purification yields and catalytic efficiency, and that these changes may be related to alterations in the secondary, tertiary, or global stability structures [10,14,39,62,63,64,65]. This could cause the clinical manifestations observed in individuals with G6PD deficiency.

## 8. Conclusions

Glucose-6-phosphate dehydrogenase (G6PD) deficiency is the most frequent human enzymopathy, affecting over 500 million people globally. Worldwide, 217 genetic mutations have been reported, and only 21 have been found in Mexico. Although all the mutations are located in different sites of the protein, they induce a decrease in catalytic efficiency, greater sensitivity to temperature denaturation, and the exposure of hydrophobic pockets in the variants, probably as the result of the less compact 3D structure of the protein. Furthermore, these mutant proteins are particularly susceptible to proteolysis or other types of damage in the half-lives of red blood cells. Finally, all the results analyzed in this review indicate that the protein dysfunction observed in G6PD-deficient individuals can be attributable to a reduction in the catalytic activity and protein instability of the G6PD mutation.

## Figures and Tables

**Table 1:** Main data about G6PD variants identified in Mexico.

Mutation Name	cDNA Nucleotide Substitution	Codon	Amino Acid Substitution	Exon	Class	Country/Region of Origin	Reference
Zacatecas	770 G>T	257	Arg → Leu	7	I	Mexico	[32]
Veracruz	1094 G>A	365	Arg → His	10	I	Mexico	[32]
Guadalajara	1159 C>T	387	Arg → Cys	10	I	Mexico, USA	[33]
Nashville	1178 G>A	393	Arg → His	10	I	USA, Italy, Portugal	[34,35]
Yucatan	1285 A>G	429	Lys → Gly	10	I	Mexico	[32]
San Luis Potosi	376 A>T	126	Asn→Tyr	5	II		[32]
Vanua Lava	383 T>C	128	Leu → Pro	5	II	Southwestern Pacific	[36]
Valladolid	406 C>T	136	Arg → Cys	5	II	Spain	[37]
Belem	409 C>T	137	Leu → Phe	5	II	Greece	[21]
Akrokorinthos	463 C>G	155	His → Asp	5	II	Brazil	[38]
Toluca	551 C>G	184	Ser → Cys	6	II	Mexico	[39]
Mediterranean	563 C>T	188	Ser → Phe	6	II	Mediterranean	[5]
Tainan	585 G>C	195	Gln → His	6	II	Taiwan/Mexico	[39,40]
Seattle	844 G->C	282	Asp → His	8	II	USA, Italy	[41,42,43]
Viangchan	871 G>A	291	Val → Met	9	II	China	[44,45]
Santa Maria	376 A>G 542 A>T	126 181	Asn → Asp Asp → Val	5, 6	II	Costa Rica, Italy	[46]
Mexico City	680 G>A	227	Arg → Gln	7	III	Mexico	[26,32]
A− (968) Betica, Selma	376 A>G 968 T>C	126 323	Asn → Asp Leu → Pro	5 9	III	Africa, Spain	[32,47]
A+	376 A>G	126	Asn → Asp	5	IV	Africa	[28,48]
Mexico DF	193 A>G	65	Ala → Thr	4	NR	Mexico	[49]

NR: class not reported.

**Table 3:** Inactivation assays for the WT and G6PD variants present in the Mexican population in the absence or presence of NADP^+^ molecule.

Enzyme	*T*_50_ (°C) without NADP^+^ (°C)	*T*_50_ with 500 µM NADP^+^ (°C)	Gdn-HCl IC_50_ (mM)	Tm	IF (-fold)	EF (-fold)	References
WT	48	59	310	59	1	1	[10]
**Class I**							
Durham	40 ↓	42 ↓	ND	50 ↓	4.4 ↑	12.8 ↑	[62]
Zacatecas	41 ↓	51 ↓	100 ↓	46 ↓	5.9 ↑	6.2 ↑	[63]
Veracruz	47 ≅	57 ↓	80 ↓	50 ↓	1.3 ↑	4.1 ↑	[14]
Guadalajara	43 ↓	47 ↓	250 ↓	50 ↓	1.3 ↑	11.6 ↑	[65]
Nasville	44 ↓	47 ↓	ND	50 ↓		6.5 ↑	[10]
Yucatán	46 ↓	51 ↓	ND	53 ↓	5.1 ↑	2.5 ↑	[10]
**Class II**							
San Luis Potosi	47 ≅	55 ↓	170 ↓	54 ↓	1 =	8.7 ↑	[65]
Vanua Lava	46 ↓	44 ↓	200 ↓	48 ↓	4.4 ↑	1.3 ↑	[63]
Valladolid	42 ↓	55 ↓	ND	53 ↓	3.9 ↑	4.4 ↑	[10]
Toluca	46 ↓	53 ↓	ND	ND	ND	ND	[39]
Tainan	43 ↓	52 ↓	ND	ND	ND	ND	[39]
Seattle	40 ↓	55 ↓	13 ↓	45 ↓	1.2 ≅	2.8 ↑	[14]
Viangchan	40 ↓	54 ↓	150 ↓	53 ↓	3.6 ↑	1.5 ↑	[63]
Santa Maria			ND	54 ↓	3.1 ↑	7.4 ↑	[62]
**Class III**							
A−	43 ↓	51 ↓	100 ↓	51 ↓	3.5 ↑	3.0 ↑	[64]
Mexico City	45 ↓	54 ↓	ND	53 ↓	5.9 ↑	10.1 ↑	[10]
**Class IV**							
A+	45 ↓	54 ↓	200	55 ↓	1.4 ↑	6.6 ↑	[64]
**Unclassified**							
Mexico DF	40 ↓	54 ↓	9 ↓	47 ↓	1.4 ↑	4.4 ↑	[14]

Tm = temperature media; IF = intrinsic fluorescence; EF = extrinsic fluorescence. ↑ Increased. ↓ Decreased. ≅ Unchanged.

## Data Availability

Not applicable.
